# The redox-sensing protein Rex modulates ethanol production in *Thermoanaerobacterium saccharolyticum*

**DOI:** 10.1371/journal.pone.0195143

**Published:** 2018-04-05

**Authors:** Tianyong Zheng, Anthony A. Lanahan, Lee R. Lynd, Daniel G. Olson

**Affiliations:** 1 Department of Biological Sciences, Dartmouth College, Hanover, New Hampshire, United States of America; 2 BioEnergy Science Center, Oak Ridge, Tennessee, United States of America; 3 Thayer School of Engineering, Dartmouth College, Hanover, New Hampshire, United States of America; 4 Center for Bioenergy Innovation, Oak Ridge, Tennessee, United States of America; University of Massachusetts, UNITED STATES

## Abstract

*Thermoanaerobacterium saccharolyticum* is a thermophilic anaerobe that has been engineered to produce high amounts of ethanol, reaching ~90% theoretical yield at a titer of 70 g/L. Here we report the physiological changes that occur upon deleting the redox-sensing transcriptional regulator Rex in wild type *T*. *saccharolyticum*: a single deletion of *rex* resulted in a two-fold increase in ethanol yield (from 40% to 91% theoretical yield), but the resulting strains grew only about a third as fast as the wild type strain. Deletion of the *rex* gene also had the effect of increasing expression of alcohol dehydrogenase genes, *adhE* and *adhA*. After several serial transfers, the ethanol yield decreased from an average of 91% to 55%, and the growth rates had increased. We performed whole-genome resequencing to identify secondary mutations in the Δ*rex* strains adapted for faster growth. In several cases, secondary mutations had appeared in the *adhE* gene. Furthermore, in these strains the NADH-linked alcohol dehydrogenase activity was greatly reduced. Complementation studies were done to reintroduce *rex* into the Δ*rex* strains: reintroducing *rex* decreased ethanol yield to below wild type levels in the Δ*rex* strain without *adhE* mutations, but did not change the ethanol yield in the Δ*rex* strain where an *adhE* mutation occurred.

## Introduction

*Thermoanaerobacterium saccharolyticum* is a thermophilic anaerobe that naturally produces ethanol. Wild type *T*. *saccharolyticum* produces ethanol at about 46% of the theoretical maximum yield [1], it also generates other fermentation products such as lactate and acetate. *T*. *saccharolyticum* has been engineered to produce ethanol at ~90% theoretical maximum yield and a titer of 70 g/L [2,3]; in these engineered strains the lactate and acetate production pathways have been deleted. While *T*. *saccharolyticum* is able to consume many of the sugars present in the hemicellulose fraction of lignocellulose, it is unable to consume cellulose. The organism has been studied both for its high levels of ethanol production and as a co-culture partner for a cellulolytic organism (e.g. *Clostridium thermocellum*) [4]. Numerous studies have focused on the roles of enzymes in *T*. *saccharolyticum*, including secreted hydrolases involved in the degradation of hemicellulose [5], the bifunctional alcohol dehydrogenase AdhE [6], and alcohol dehydrogenase AdhA [1]. Additionally, fermentation end-product analyses [7] and genome-scale microarray data [8] have been reported.

In addition to studying the central metabolic pathways in *T*. *saccharolyticum*, we are also interested in the regulation of genes involved in the ethanol production pathway. Rex is a global transcription factor that has been studied in many facultative [9–12] and strict [13–19] anaerobes. It senses intracellular NADH/NAD^+^ levels and controls the expression of many genes involved in energy metabolism and anaerobic fermentation. In its homodimer form, Rex represses gene expression by binding to both the promoter region of a DNA strand (N-terminus) and an NAD^+^ molecule (C-terminus) thus inhibiting transcription of the target gene. When the molecule of NAD^+^ bound to its C-terminus is replaced by NADH, conformational changes trigger the release of the Rex dimer from the DNA thereby allowing transcription to proceed [20]. The Rex protein is important in regulating metabolism: when NADH/NAD^+^ ratios are high in the cell, it signals that energy-generating catabolic processes (such as glycolysis) are proceeding at a sufficient rate. Therefore by releasing *adhE* repression under high concentrations of NADH, AdhE can convert NADH to NAD^+^ and replenish the NAD^+^ pool.

We have recently identified key alcohol dehydrogenase genes in the *T*. *saccharolyticum* ethanol production pathway: *adhE* and *adhA* are both necessary for high levels of ethanol production in *T*. *saccharolyticum* [1]. *In vitro* assays have shown that Rex regulates *adhE* expression in *Staphylococcus aureus* [10], *Streptococcus mutans* [11,21], *Enterococcus faecalis* [12], *Thermoanaerobacter ethanolicus* [13], *Clostridium acetobutylicum* [14,15], and *Thermotoga* sp. RQ-2 [16].

Moreover, Rex has previously been inactivated in *S*. *aureus* [10], *E*. *faecalis* [12], *C*. *acetobutylicum* [14,15], *Desulfovibrio vulgaris* [17], and *S*. *mutans* [21]. In *C*. *acetobutylicum* and *S*. *aureus*, ethanol yield increased in the Rex-inactivated strains [10,14,15]. These results suggest that inactivating Rex could serve as an engineering strategy to increase desired fermentation products such as ethanol. Therefore one aim of this study was to examine the role of Rex in *T*. *saccharolyticum* ethanol production. Another aim was to explore the potential of deleting *rex* as an engineering strategy for higher ethanol yield in bacterial organisms. Specifically, we were interested in whether deleting *rex* would create a high-ethanol phenotype that is stable across numerous generations. A final aim was to identify putative Rex-binding sites in *T*. *saccharolyticum*, to better understand which genes it regulates.

## Materials and methods

### Plasmid and strain construction

Strains and cloning plasmids used in this study are listed in Table 1. The *rex* deletion was made with pTZvec13 using previously-described techniques for genetic modification [1,22]. Briefly, pTZvec13 consists of a kanamycin marker flanked by the upstream and downstream regions of the *rex* gene. Sequence information of pTZvec13 can be found in Genbank accession number KY863518. *T*. *saccharolyticum* cells and the pTZvec13 vector were inoculated into a rich growth medium (CTFUD) [23] and incubated at 55 °C to allow transformation (note that *T*. *saccharolyticum* is naturally competent [22]). Cells were harvested at OD_600_ ~0.6 and plated on CTFUD agar with 200 μg/ml kanamycin. Colonies were picked following a two-day incubation period at 55 °C and subsequently analyzed by PCR and gel electrophoresis. Kanamycin was only used in the medium during the initial strain isolation process to select for the chromosomal gene deletion. Deletion of *rex* was confirmed by PCR and whole-genome sequencing.

**Table 1:** Strains used in this study.

Strain name	Description	Cloning plasmids	Source or reference
LL1025	Wild-type *T*. *saccharolyticum* strain JW/SL-YS485	/	Mai *et al*. [24] Genbank No. CP003184
LL1414	Wild-type *T*. *saccharolyticum* Δ*rex*::*kan* strain, a.k.a. Rex2	pTZvec13	This study. Genbank No. KY863518
LL1415	Wild-type *T*. *saccharolyticum* Δ*rex*::*kan* strain, a.k.a. Rex4	pTZvec13	This study. Genbank No. KY863518
LL1416	Wild-type *T*. *saccharolyticum* Δ*rex*::*kan* strain, a.k.a. Rex5	pTZvec13	This study. Genbank No. KY863518
LL1417	Wild-type *T*. *saccharolyticum* Δ*rex*::*kan* strain, a.k.a. Rex8	pTZvec13	This study. Genbank No. KY863518
LL1356	Rex2 after serial transfers, a.k.a. RexAdp-2	/	This study. SRA No. SRP112528
LL1357	Rex4 after serial transfers, a.k.a RexAdp-4	/	This study. SRA No. SRP112529
LL1358	Rex5 after serial transfers, a.k.a RexAdp-5	/	This study. SRA No. SRP112523
LL1359	Rex8 after serial transfers, a.k.a RexAdp-8	/	This study. SRA No. SRP11252
LL1553	LL1357 rex complementation strain, a.k.a RexCmp-4	pTZvec14	This study. Genbank No. MG020537
LL1554	LL1358 rex complementation strain, a.k.a RexCmp-5	pTZvec14	This study. Genbank No. MG020537

The *rex* gene deletion was complemented using the plasmid pTZvec14 (Genbank accession number MG020537), which consists of the *rex* gene and an erythromycin marker, flanked by upstream and downstream regions of the *rex* deletion region. The transformation protocol of pTZvec14 is identical to that of pTZvec13 as described above, followed by incubation at 50 °C with 15 μg/ml erythromycin selection. Resulting complementation strains are referred to as RexCmp-4 and RexCmp-5 (Table 1). Sanger sequencing of the reconstituted *rex* region in strains RexCmp-4 and RexCmp-5 confirmed successful complementation of the gene.

### Media and growth conditions

*T*. *saccharolyticum* strains were grown in CTFUD rich medium for transformations and enzymatic assays as previously described [6]. Following transformation, *rex* deletion colonies #2,4,5 and 8 were picked and inoculated into CTFUD rich medium, resulting in strains Rex-2, Rex-4, Rex-5 and Rex-8 (a.k.a LL1414-1417 in Table 1). These strains were then subjected to 3~7 rounds of serial transfers (1% v/v inoculum) on a chemically defined medium MTC-6 [25]. Specifically, strain Rex-2 underwent 6 rounds of serial transfers (~40 generations) and became strain RexAdp-2 (a.k.a. LL1356); Rex-4 and Rex-5 underwent 3 rounds of serial transfers (~20 generations) and became strains RexAdp-4 and RexAdp-5 (a.k.a. LL1357 and LL1358 respectively); Rex-8 underwent 7 rounds of serial transfers (~46 generations) and became strain RexAdp-8 (a.k.a. LL1359). To promote cell growth for use in fermentation end product analysis, RT-qPCR, and growth-rate analysis, *T*. *saccharolyticum* strains were grown in MTC-6 medium with the addition of 0.5 g/L yeast extract (a.k.a “modified MTC-6 media”).

### Fermentation end product analysis and enzymatic assays

For fermentation end product analysis, strains were inoculated into 5 ml modified MTC-6 media (5 g/L cellobiose) at 1% v/v inoculum. Cultures were grown in Corning^TM^ Falcon^TM^ 15 ml Conical Centrifuge Tubes and incubated anaerobically without shaking at 55 °C for 72 hours. Each fermentation experiment was performed in biological duplicates. Upon harvesting, cultures were prepared as previously described for HPLC (High Pressure Liquid Chromatography) analysis [1]. Fermentation end products are presented in S1 Table.

For enzymatic assays, cell extracts were generated and assays were performed as previously described [1]. Briefly, ADH reactions were performed anaerobically at pH 7.0, 55°C, in a reaction mixture containing 0.2 mM NADH or NADPH, 20 mM acetaldehyde, 100 mM Tris-HCl, 5 μM FeSO_4_ and different concentrations of cell extract. The final volume was 1000 μl, and the reaction was initiated by addition of acetaldehyde. ADH activity was measured in the ethanol-forming direction; one unit of activity (U) is described as the formation of 1 μmol of product per minute. Specific activities are expressed as U/mg of protein. Protein concentrations of cell extracts were measured using the Pierce^TM^ Bradford Assay Kit (part number 3200) with bovine serum albumin as the standard.

### Growth rate analysis

Sterile 96-well flat-bottom plates were used to culture strains for growth rate analysis. Each well was filled with 200 μL modified MTC-6 medium and inoculated with actively growing cultures to a starting OD_600_ of ~0.05. The plate was sealed with a transparent sealing film and placed into a BioTek PowerWave XS plate reader inside an anaerobic chamber. The plate was shaken for 30 seconds every 3 minutes, followed by an OD_600_ measurement. The incubation temperature of the plate reader was 55°C and cultures were grown for three days. OD_600_ values at each time point were exported to GraphPad Prism 7.0a, where growth curves were generated and growth rates were calculated.

### Gene expression analysis

RT-qPCR was performed to measure gene expression. Cells were grown at 55°C to mid-log phase—OD_600_ ~ 0.5 for faster growing strains (wild type, LL1356-1359), and OD_600_ ~ 0.2 for slower growing strains (LL1414-1417). Harvested cultures were stored at -20°C after addition of RNAprotect (Qiagen). Genomic RNA was prepared using the RNeasy Mini Kit (Qiagen part number 74104), and subsequent cDNA was synthesized using the iScript cDNA Synthesis Kit (BioRad part number 170–8891). To assure adequate removal of the genomic RNA, control reactions were performed where reverse transcriptase was not added. RT-qPCR was performed using the SsoFast^™^ EvaGreen^®^ Supermix (BioRad part number 172–5201). For quantification, a gBlock template that includes the *adhE*, *adhA* and *recA* amplicons sequences in a 1:1:1 ratio was diluted to known concentrations to generate a standard curve. Primers used for qPCR are presented in S2 Table. Standard molecular techniques were used to perform the RT-qPCR reaction; biological duplicates and technical triplicates were performed for each reaction.

### Whole genome sequencing

Strains LL1356-1359 were grown on CTFUD rich medium (note that CTFUD medium gives better yields of genomic DNA yield, compared with MTC-6). The genomic DNA was submitted to the Joint Genome Institute (JGI) where Illumina MiSeq sequencing was performed as previously described [25], generating paired-end reads with an average read length of 150 bp and paired distance of 500 bp. Raw data was analyzed using CLC Genomics Workbench, version 8 (Qiagen, USA). First reads were mapped to the reference genome (NC_017992). Mapping was improved by 2 rounds of local realignment. The CLC Probabilistic Variant Detection algorithm was used to determine small mutations (single and multiple nucleotide polymorphisms, short insertions and short deletions). Variants that were identical to those of the wild type strain (i.e. due to errors in the reference sequence) were filtered out.

To determine larger mutations, the CLC InDel and Structural Variant algorithm was run. This tool analyzes unaligned ends of reads and annotates regions where a structural variation may have occurred, which are called breakpoints. Since the read length averaged 150 bp and the minimum mapping fraction was 0.5, a breakpoint can have up to 75 bp of sequence data. The resulting breakpoints were filtered to eliminate those with fewer than 10 reads or less than 20% “not perfectly matched.” The breakpoint sequence was searched with the Basic Local Alignment Search Tool (BLAST) algorithm [26] for similarity to known sequences. Pairs of matching left and right breakpoints were considered evidence for structural variations such as transposon insertions and gene deletions.

### Prediction of Rex-regulated genes

The Rex binding motif was searched for in the *T*. *saccharolyticum* genome using the web-based tool PATLOC (http://www.cmbl.uga.edu/software/patloc.html) [27]. A palindromic sequence consisting of 5 AT rich nucleotides separated by 8 AT-rich nucleotides was used to search for the Rex-binding motif, syntax codes WTGWW{WWWWWWWW}[1]-5-4-3-2-1, WTGWW{WWWWWWWW}[1]-5-4-3-N-1 and WTGWW{WWWWWWWW}[1]-5-4-3-2-N were used as the search criteria. This search criteria yielded palindromic sequences allowing at most one mismatch between the 5 bp AT-rich sequence and its reverse complementary sequence occurring within the last two nucleotides (5-4-3-N-1 and 5-4-3-2-N). The search criteria also allowed at most one C/G nucleotide in the 8 bp AT-rich span. Both strands of DNA in the genome were included in the search. Putative binding sites that were more than 200 bp upstream of the nearest gene or where the function of the nearest gene was not annotated were excluded from subsequent analysis.

## Results

### Deleting *rex* increased ethanol yield but decreased growth rate

**Fig 1:**
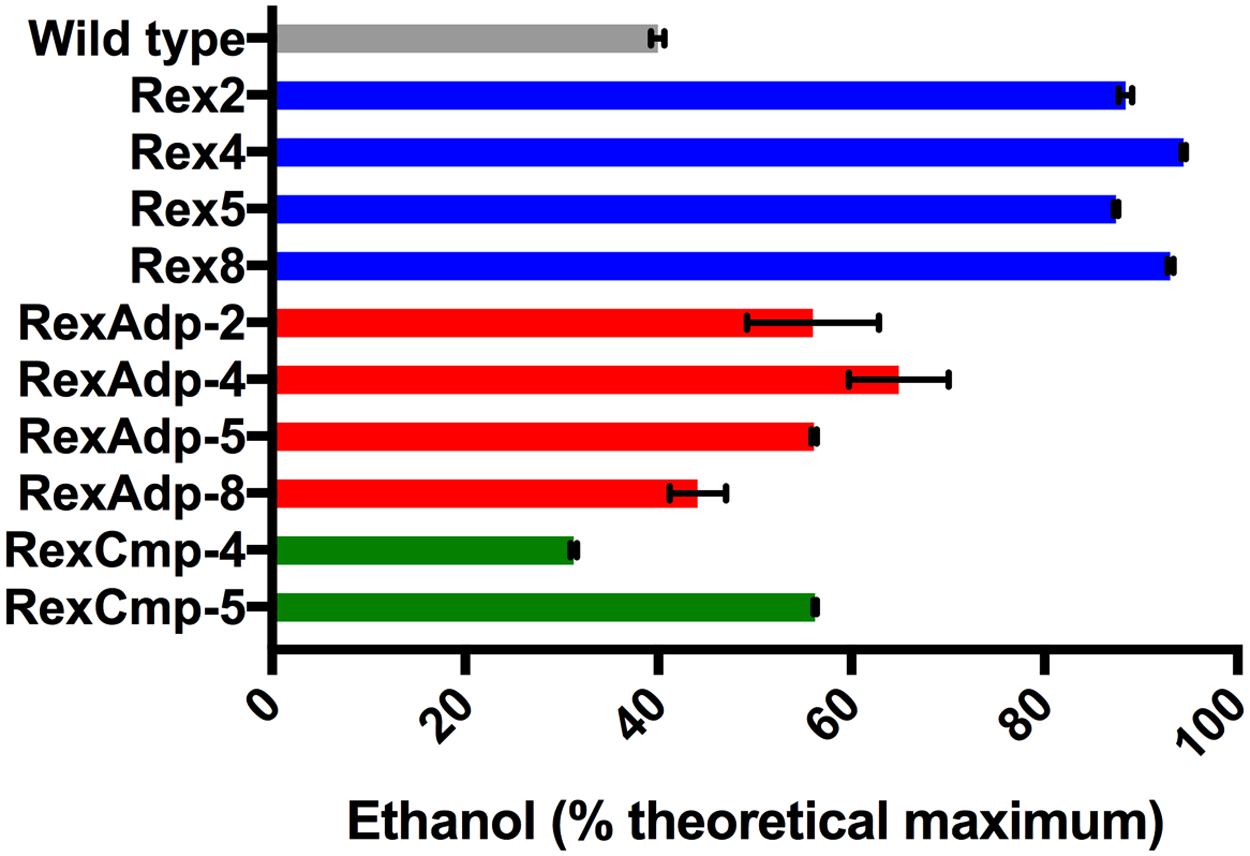
Ethanol yields of Δ*rex* strains before and after serial transfers. Wt is shown in grey, Δ*rex* strains before adaptation are shown in blue, Δ*rex* strains after adaptations in red, and *rex* complementation strains in green. Cultures were grown on 5 g/L cellobiose in modified MTC-6 media (see Material and methods), and theoretical ethanol yield from cellobiose is 0.54 g ethanol per gram cellobiose. Ethanol yields are calculated based on the amount of substrate consumed (initial and final concentrations of cellobiose were measured). Ethanol yield is presented in percent theoretical maximum, which assumes that one molecule of glucose (or glucose equivalent) can be converted into, at most, two molecules of ethanol.

In wild type *T*. *saccharolyticum*, the Tsac_2615 gene encodes a protein with 87% amino acid sequence identity to the *T*. *ethanolicus* Rex protein. We deleted the *T*. *saccharolyticum rex* gene (Tsac_2615) by replacing it with the kanamycin resistance gene *kan* (see Material and methods), resulting in strains Rex-2, Rex-4, Rex-5 and Rex-8 (each representing a single colony). These strains produced ethanol at an average of 91% theoretical maximum yield, which was more than a two-fold increase compared to wild type (40%) (Fig 1). However, growth rates significantly decreased in these Δ*rex* strains and each Δ*rex* strain had a μ_MAX_ that was less than a third of that in wild type (Table 2).

**Table 2:** Growth rate and Max OD_600_ of Δ*rex* strains.

Strain	Growth rate μ_MAX_	Max OD_600_
wild type	0.31 ± 0.01	0.43 ± 0.02
Rex2	0.10 ± 0.01	0.44 ± 0.02
Rex2-Adp	0.38 ± 0.02	0.76 ± 0.01
Rex4	0.09 ± 0.01	0.29 ± 0.01
Rex4-Adp	0.29 ± 0.01	0.68 ± 0.01
Rex5	0.06 ± 0.07	0.20 ± 0.02
Rex5-Adp	0.16 ± 0.01	0.61 ± 0.02
Rex8	0.06 ± 0.01	0.29 ± 0.03
Rex8-Adp	0.12 ± 0.01	0.24 ± 0.01

### Adaptation increased growth rate but decreased ethanol yield in Δ*rex* strains

To investigate the stability of the high ethanol phenotype in the Δ*rex* strains, we conducted serial transfers for strains Rex-2, 4, 5 and 8 (see Material and methods). After at least 20 generations, the adapted strains (RexAdp-2, RexAdp-4, RexAdp-5, and RexAdp-8) all grew faster than their parent strains, displaying a two to three-fold increase in growth rate (Table 2, μ_MAX_). Three out of four adapted strains (RexAdp-2, 4, 5) had higher maximal OD_600_ levels than their parent strains (Table 2). Interestingly, this increase in growth rate was accompanied by a 29%-50% decrease in ethanol yield comparing to non-adapted Δ*rex* strains (Fig 1).

### Secondary mutations in adapted Δ*rex* strains

Adapted strains RexAdp-2, RexAdp-4, RexAdp-5, and RexAdp-8 were submitted for whole-genome sequencing to analyze secondary mutations associated with the *rex* deletion. Mutations in the DNA sequence that caused alterations in amino acid sequences or promoter regions were found at 27 locations across the genome, as listed in Table 3. Most mutations occurred in transcription and biosynthesis related genes, as well as genes encoding membrane-associated proteins. Most of these mutations only occurred in one of the four adapted strains, but mutations in *adhE* (Tsac_0416), *trx* (Tsac_0492), Auxin efflux carrier (Tsac_2140), and *rpoN* (Tsac_2488) appeared frequently, appearing in at least three out of four adapted strains. In particular, *adhE* accumulated three different mutations, as well as a mutation in its promoter region (Table 3).

**Table 3:** Secondary mutations in adapted Δ*rex* strains.

Locus/ location	Gene	Protein product of locus gene or downstream gene	Category	Start site	Type^a^	Mutation description ^b^	Fraction of reads supporting mutation^c^			
							RexAdp-2	RexAdp-4	RexAdp-5	RexAdp-8
Tsac_0038		UbiC transcription regulator-associated	Trans ^d^	37942	SNV	G → A, Leu49Phe	48%	0%	0%	0%
Tsac_0138	*araC*	Two component transcriptional regulator	Trans	143079	INS	*T*. *saccharolyticum* mega plasmid CP003185	0%	0%	95%	0%
Tsac_0377		ACT domain-containing protein	Syn	408737	SNV	C → A, Gly67Trp	99%	0%	0%	0%
Tsac_0393		CRISPR-associated protein	Misc	430181	SNV	C → A, Glu454*	0%	0%	99%	0%
184 bp upstream of Tsac_0416	*adhE*	AdhE, Alcohol dehydrogenase, iron-type	Carb	447044	INS	- → AT	0%	38%	0%	20%
Tsac_0416				449017	SNV	C → A, Thr597Lys	57%	0%	0%	0%
					SNV	C → T, Thr597Ile	30%	0%	0%	0%
				449041	SNV	C → T, Thr605Ile	10%	0%	76%	0%
Tsac_0492	*trx*	Pyridine nucleotide-disulphide oxidoreductase	Misc	517443	SNV	T → C, Lys27Arg	100%	100%	0%	100%
Tsac_0542		Cobyrinic acid a,c-diamide synthase	Syn	566929	SNV	G → T	0%	0%	0%	100%
Tsac_0947		Putative transcriptional activator, Baf family	Trans	992952	SNV	T → C	0%	0%	100%	0%
Tsac_1176		Histidinol dehydrogenase	Syn	1219174	SNV	C → T, Pro369Ser	0%	0%	100%	0%
Tsac_1265		Selenoprotein B	Syn	1315478	SNV	T → C	0%	0%	67%	0%
Tsac_1588	*phoU*	PhoU	Mem	1667093	SNV	C → T, Gln71*	0%	0%	84%	0%
Tsac_1705		Dihydroorotate dehydrogenase	Syn	1775863	INS	*T*. *saccharolyticum* mega plasmid CP003185	0%	0%	90%	0%
Tsac_1961	*sigK*	RNA polymerase, sigma 28 subunit	Trans	2009358	INS	*T*. *saccharolyticum* phage THSA-485A, CP003186	0%	0%	81%	0%
46 bp upstream of Tsac_2008		Peroxiredoxin	Misc	2054419	SNV	C → T	0%	0%	100%	0%
32 bp upstream of Tsac_2140		Auxin efflux carrier family protein	Mem	2178994	DEL	ATA → -	100%	100%	0%	100%
Tsac_2140				2179432	SNV	C → T, Ser135Leu	0%	0%	100%	0%
Tsac_2480		Uncharacterized protein family UPF0251	Misc	2530745	INS	Possible transposon downstream of Tsac_1830	0%	57%	0%	0%
Tsac_2488	*rpoN*	RNA polymerase, sigma 54 subunit	Trans	2538897	SNV	C → A, Glu395*	0%	0%	82%	0%
					SNV	C →A, Glu395Gln			20%	
				2539210	DEL	T → -, Lys290fs	0%	0%	0%	99%
				2539210	INS	- → TTTTTGTATTGTCTTTGACAAGTA, Lys289_Lys290insAsnThrCysGlnArgGlnTyrLys	97%	0%	0%	0%
Tsac_2564		phosphotransferase system PTS lactose/cellobiose-specific IIA subunit	Mem	2618525	SNV	C → T, Ala80Thr	0%	0%	100%	0%
Tsac_2602		Extracellular ligand-binding receptor	Mem	2652896	INS	- → AAT, Asn85_Lys86insAsn	0%	87%	0%	0%
Tsac_2615	*rex*	Redox-sensing transcriptional repressor	Trans	2663911	Targeted insertion	rex gene replaced with kan	100%	100%	100%	100%

^a^ SNV: single nucleotide variation; INS: insertion; DEL: deletion

^b^ * annotates a stop codon,—annotates a deletion

^c^ percentage of sequencing reads that contain mutation. Variants that were identical to those of the wild type strain were filtered out.

^d^ Trans: transcription; Syn: biosynthesis; Misc: miscellaneous; Carb: carbon metabolism; Mem: membrane-associated

### *adhE* and *adhA* expression levels increased in Δ*rex* strains

**Fig 2:**
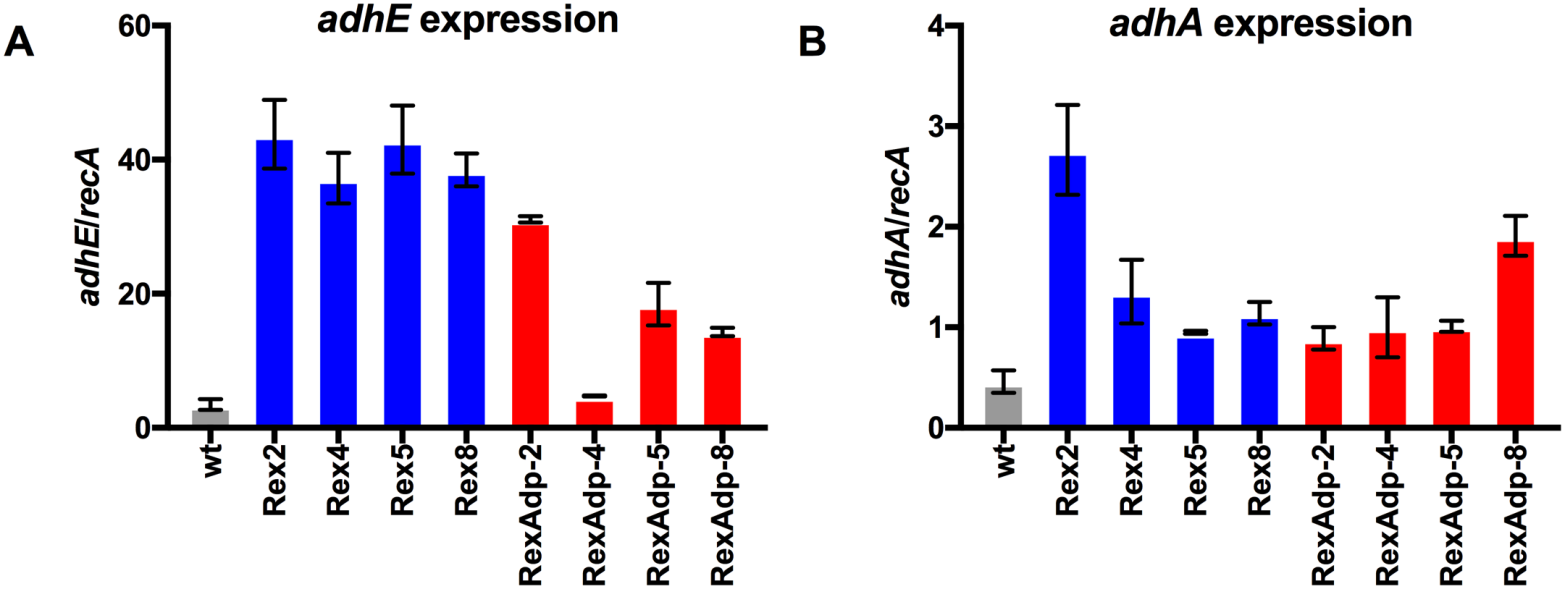
Gene expression levels of *adhE* and *adhA*. Panel A shows *adhE* expression levels and panel B shows *adhA* expression levels. Strains were grown at 55°C to mid-log phase—OD_600_ ~ 0.5 for faster growing strains (wild type, LL1356-1359), and OD_600_ ~ 0.2 for slower growing strains (LL1414-1417). Wt is shown in grey, Δ*rex* strains before adaptation in blue and Δ*rex* strains after adaptation in red. Gene expression levels of *adhE* and *adhA* are normalized to *recA*. Biological duplicates were performed for each reaction; error bars represent one standard deviation.

Gene expression levels of *adhE* and *adhA* were measured via RT-qPCR and results are presented in Fig 2. When *rex* was deleted in wild type *T*. *saccharolyticum*, the resulting strains Rex-2, 4, 5, 8 showed a ten to twelve-fold increase in *adhE* expression and two to six-fold increase in *adhA* expression (Fig 2). However, after adaptation, the resulting strains RexAdp-2, 4, 5, 8 showed significant decreases in *adhE* expression comparing to non-adapted Δ*rex* strains (Rex-2, 4, 5, 8). Nevertheless, *adhE* expression in the adapted strains was still higher than wild type levels (Fig 2, panel A). *adhA* expression levels in the adapted strains were similar or slightly higher than those in non-adapted Δ*rex* strains (Fig 2, panel B).

### Alcohol dehydrogenase activity increased in Δ*rex* strains

To determine the effect of the *rex* deletion on alcohol dehydrogenase (ADH) enzymatic activity, ADH activity in the direction of ethanol formation was measured. As presented in Table 4, comparing to wild type, NADH-linked ADH activity increased when *rex* was deleted (Rex-2, 4, 5, 8). The adapted strains RexAdp-2, 4, 5, and 8 showed different levels of decrease in NADH-ADH activity compared to the non-adapted strains. RexAdp-2 and RexAdp-5 showed almost complete elimination of NADH-ADH activity. This was not unexpected, as both RexAdp-2 and RexAdp-5 had at least one mutation in *adhE* (Table 3). Surprisingly, we did not see an increase in NADPH-ADH activity (contributed by AdhA) in either the adapted or the non-adapted Δ*rex* strains (Table 4). In fact, NADPH-ADH activity remained at about 50% of wild-type levels in all measured Δ*rex* strains (Table 4).

**Table 4:** ADH activity in Δ*rex* strains.

Strain	LL number	Description	ADH specific activity ^a^		Ethanol yield (% theoretical) ^b^
			NADH	NADPH	
Wild type	LL1025	Wild type	2.39 ± 0.01	0.77 ± 0.16	40%
Rex-2	LL1414	Before adaptation	4.89 ± 0.37	0.31 ± 0.04	88%
RexAdp-2	LL1356	After adaptation, mutation in *adhE*	0.00 ± 0.05	0.24 ± 0.01	56%
Rex-4	LL1415	Before adaptation	5.17 ± 0.77	0.33 ± 0.1	94%
RexAdp-4	LL1357	After adaptation, mutation upstream of *adhE*	3.43 ± 0.84	0.25 ± 0.07	65%
Rex-5	LL1416	Before adaptation	11.23 ± 1.96	0.35 ± 0.09	87%
RexAdp-5	LL1358	After adaptation, mutation in *adhE*	0.02 ± 0.01	0.31 ± 0.06	56%
Rex-8	LL1417	Before adaptation	13.23 ± 1.64	0.36 ± 0.06	93%
RexAdp-8	LL1359	After adaptation, mutation upstream of *adhE*	2.42 ± 0.28	0.28 ± 0.04	44%

^*a*^ Specific activity is an average of two technical replicates. Error is one standard deviation. Units are U/mg cell extract protein, where 1 U indicates the formation of 1 umol of product per minute.

^*b*^ ethanol yields from Fig 1, provided here for comparison.

### Complementation of the *rex* deletion

To separate the effect of deleting *rex* from the effect of subsequent secondary mutations, we complemented the *rex* deletion by inserting *rex* at its original locus (see Material and methods). We hypothesized that if the secondary mutations did not affect the regulatory role of Rex in ethanol production, then complementation of *rex* in the adapted Δ*rex* strains should result in an ethanol yield comparable to or lower than wild type levels. We performed the complementation in all four of the adapted strains RexAdp-2, 4, 5, 8, however only RexAdp-4 and RexAdp-5 yielded successful transformants: RexCmp-4 and RexCmp-5. This is possibly due to the lower transformation efficiency we observed with erythromycin selection in *T*. *saccharolyticum* as compared to kanamycin selection. Analysis of fermentation end products showed that strain RexCmp-4 produced ethanol at 31% theoretical yield, which was lower than wild type levels and about half the ethanol yield in its parent strain. On the contrary, RexCmp-5 produced ethanol at 56% theoretical yield, which was the same as its parent strain RexAdp-5.

### Predicted Rex-binding sites in *T*. *saccharolyticum*

We selected the Rex-binding DNA motif ATTGTTANNNNNNTAACAAT in *T*. *ethanolicus* [13] as a template to search for similar motifs in *T*. *saccharolyticum*, due to the close phylogenic relationship between the two organisms. The *T*. *ethanolicus* Rex-binding DNA motif was searched using the BLAST algorithm against the upstream regions of *adhE* and *adhA* of *T*. *saccharolyticum*, and a putative *T*. *saccharolyticum* Rex-binding DNA motif with a consensus sequence of TTGTTANNNNNNTAACNN was identified (S1 Fig). Based on this consensus sequence, we searched for similar palindromes comprising 5 bp inverted repeats with a G/C center (e.g. TTGTT and AACAA), where the inverted repeats were separated by an 8 bp span of AT-rich bases (see Material and methods). The potentially functional binding sites (within 200 bp of the nearest downstream gene) are summarized in Table 5. Many predicted sites were located upstream of genes related to energy metabolism, carbon metabolism, biosynthesis, and membrane-associated transporters.

**Table 5:** Putative Rex-binding sites in *T*. *saccharolyticum*.

Locus ^a^	Gene(s) ^b^	Protein product	Category	Rex-binding sequence	Distance to gene ^c^
Tsac_0014		Cof-like hydrolase	Misc ^d^	TTGTAAATTTACATACTA	-65
Tsac_0416	*adhE*	AdhE	Carb	TTGTTAAATGAATAACAA	-150
Tsac_0416	*adhE*	AdhE	Carb	TTGTTAATAAATTAACAC	-31
Tsac_0648		Cation diffusion facilitator family transporter	Mem	ATGATAATCAATAATCTT	-116
Tsac_0675	*echABCDEF-hypABFCDE*	Energy-conserving Ni-Fe hydrogenase; Ni-Fe hydrogenase maturation factor	Energy	TTGTTAAATAATAAACAA	-138
Tsac_0692		Extracellular solute-binding protein family 1	Mem	TTGAAATTATTATTTCAA	-169
Tsac_0706	*asrABC*	Anaerobic sulfite reductase	Misc	TGGTTATTTATTTAACAAA	-56
Tsac_0744		Stage V sporulation protein T	Spo	TTGAACTTAAAATTTCAG	-36
Tsac_0924		ACT domain-containing protein	Syn	ATGATAATAAAACATCAA	-82
Tsac_0932		Stage II sporulation protein E	Spo	ATGATATAATCATATCAA	-37
Tsac_0989		Glutamyl-tRNA synthetase	Syn	ATGTATATGATAATACAA	-35
Tsac_1035		Translation initiation factor IF-1	Syn	AAGAAAAAATTGATTCAT	-154
Tsac_1163		ABC-type transporter, integral membrane subunit	Mem	ACGATATATGTAAATCAT	-92
Tsac_1375	*marR*	Regulatory protein MarR	Misc	CTGAAATAATCATTTCAA	-86
Tsac_1550	*hfsABCD*	Iron hydrogenases; sporulation protein	Energy	TTGTTAATAAATTAACTA	-78
Tsac_1619		Prephenate dehydrogenase	Carb	GTGATTTTAAATGATCAA	-10
Tsac_1753		3-oxoacyl-(acyl-carrier-protein) reductase	Syn	TTGATAAACTTTTATCCA	-35
Tsac_1856		PHP domain protein	Syn	TTGTTTTTAGTTTAACAT	-33
Tsac_1947		Fimbrial assembly family protein	Mem	ATGATTCAAAAAAATCAA	-186
Tsac_1985		ABC transporter related	Mem	TTGAATTGAAATTTTCTA	-82
Tsac_2078		ABC-2 type transporter	Mem	CTGATGAATTAAAATCAA	-99
Tsac_2087	*adhA*	Alcohol dehydrogenase AdhA	Carb	TAGTTAAATTTATAACAA	-34
Tsac_2115	*rodA*	Rod shape-determining protein RodA	Misc	TAGTATTATATCATACAA	-60
Tsac_2192		MutS2 protein	Syn	ATGAATTTTTGTATTCTT	-88
Tsac_2363	*hydG*	Fe-hydrogenase maturation protein	Energy	TTGTTAAATATTCAACAA	-92
Tsac_2550		Nucleoside-triphosphatase rdgB	Syn	ATGATTTTATTCAATCCT	-74
Tsac_2619		Gluconate 2-dehydrogenase	Carb	TTGATTACATAATATCCA	-44
Tsac_2652		Sigma 54 modulation protein/ribosomal proteinS30EA	Misc	ATGAAATCTAATATTCCT	-62
Tsac_2683		Phosphoribosyltransferase	Syn	AAGATTATTATCTATCAT	-165

^*a*^ The locus tag of the nearest downstream gene

^*b*^ Known gene names based on sequence homology

^*c*^ Distance from Rex-binding site to the start of the nearest downstream gene (including length of the Rex-binding site)

^*d*^ Trans: transcription; Syn: biosynthesis; Misc: miscellaneous; Carb: carbon metabolism; Mem: membrane-associated; Spo: sporulation

## Discussion

### *adhE* and *adhA* gene expression increased in Δ*rex* strains

As shown in Fig 2, expression of alcohol dehydrogenase genes *adhE* and *adhA* increased in Δ*rex* strains Rex-2, 4, 5, and 8. The significant increase in *adhE* expression agrees with previous studies in other microorganisms that measured *adhE* gene expression in Rex-inactivated strains [10,12,15,21], suggesting that Rex regulates *adhE* expression in *T*. *saccharolyticum*. Corresponding to the decrease in ethanol production in adapted Δ*rex* strains (Fig 1), a decrease in *adhE* expression was also observed (Fig 2). RexAdp-4 and RexAdp-8 had the largest decrease in *adhE* expression, which may be explained by the secondary mutation found 184 bp upstream of *adhE* in these strains (Table 3).

In *T*. *ethanolicus*, *adhA* was a predicted target of Rex based on its binding motif and was shown to be regulated by Rex in *in vitro* DNA-binding assays [13]. In the current study we provide *in vivo* gene expression data that suggests Rex is a regulator for *adhA*. It should be noted that AdhA herein refers to the iron-containing alcohol dehydrogenase with at least 60% protein sequence identity to the *T*. *saccharolyticum* AdhA (as previously reviewed [1], a variety of proteins named AdhA exist that are not homologous to the *T*. *saccharolyticum* AdhA). Interestingly, the increase in *adhA* expression was less significant than the increase in *adhE* expression in Δ*rex* strains (Fig 2). This may be due to the fact that only one predicted Rex binding site was found upstream of *T*. *saccharolyticum adhA*, while two were found upstream of *adhE* (Table 5).

Taken together, the data strongly suggests that Rex negatively regulates both *adhE* and *adhA* in *T*. *saccharolyticum*.

### Secondary mutations and their effect on ethanol production

One of the most significantly affected genes by deleting *rex* was *adhE*. All of the adapted *rex* deletion strains had mutations either within or just upstream of the *adhE* gene. AdhE is a bifunctional alcohol dehydrogenase with two domains, ADH and ALDH, and it catalyzes the last two steps of ethanol formation [1,6]. One explanation for the high frequency of mutations in *adhE* would be the sudden increase in ethanol production after deleting *rex* (Fig 1). Without the ability to regulate *adhE* expression levels, the NADH pool could become depleted due to over-production of ethanol. This would be disruptive to metabolism, leading to slower growth. Additionally, overflow through the ethanol pathway would decrease the flow toward acetate production, as shown by the ~10 fold decrease in acetate production in S1 Table. Decreased acetate production would lead to a decrease in ATP production, contributing to slower growth. Therefore, *adhE* mutations that limit ethanol production could provide a selective advantage.

Protein sequence alignment showed that AdhE mutations T597K, T597I, and T605I, occurred at highly conserved residues in the alcohol dehydrogenase domain of AdhE (S2 Fig), thus we would anticipate a large decrease in ADH enzymatic activity. Indeed, in RexAdp-2 and RexAdp-5, we saw complete elimination of NADH-linked ADH activity (Table 4), which was expected because the wild type *T*. *saccharolyticum* AdhE was shown to be NADH-linked [6].

However, there were no mutations in *adhA*, a gene we predicted to be regulated by Rex as well (Table 5). Corresponding enzymatic activity also showed no significant reduction in NADPH-linked ADH activity compared to the non-adapted parent strains (Table 4), indicating no reduction in AdhA activity [1]. The lack of influence of the *rex* deletion on AdhA activity suggests a weaker control of Rex on *adhA* expression.

We have previously shown that although both NADH and NADPH-linked ADH enzymes exist in *T*. *saccharolyticum*, the actual flux through these pathways can vary [28]. Interestingly, while the electron transfer stoichiometry is significantly different in the two systems, both the NADH-linked and the NADPH-linked pathways resulted in high ethanol yield [28]. In this study, although total ADH activity (NADH+NADPH-linked) decreased by ~95% in RexAdp-2 and RexAdp-5 compared to their parent strains due to loss of AdhE ADH function, their ethanol yields only decreased by about 36% (Fig 1 and Table 4). The ability to produce ethanol indicates that the mutation did not affect the ALDH domain of AdhE, as AdhE is the only known enzyme capable of reducing acetyl-CoA to acetaldehyde, based on our current understanding [1]. The seemingly discrepancy between loss of enzyme activity and loss of ethanol yield may be due to the shift in ADH cofactor specificity. It is likely that before the *adhE* mutation, there was not a significant flux through AdhA (although the enzyme is fully functional as measured by *in vitro* assays), and the electron flux from pyruvate to acetyl-CoA mainly flows through the NADH-linked FNOR (Ferredoxin:NAD^+^ oxidoreductase) [29], rendering this a primarily NADH-linked system. After the *adhE* mutation, the cell relies completely upon the NADPH-linked AdhA for reduction of acetaldehyde to ethanol. From pyruvate to acetyl-CoA, electrons would likely flow through NfnAB (NADP oxidoreductase) instead of the NADH-linked FNOR to balance the NADP^+^ generated by AdhA, therefore shifting toward a primarily NADPH-linked system. The ability for AdhA to produce high levels of ethanol in the absence of AdhE ADH activity agrees with previous results [1][30].

Additionally, we saw several mutations accumulate in RNA polymerase genes *sigK* and *rpoN*, indicating an overall decrease in transcription. There were also mutations in biosynthesis genes and genes encoding membrane-associated proteins (Table 3). These mutations in biosynthesis genes may reflect a stress response to the sudden increase in ethanol production upon loss of *rex*. The effects of ethanol stress have been most extensively studied in yeast [31]. Ethanol stress influences several aspects of yeast physiology, resulting in decreased mRNA and protein levels [32,33], decreased transport processes [34], and decreased H^+^-ATPase activity [35]. It is also well known that ethanol has a negative effect on membrane-associated proteins [36–38]. Therefore, the secondary mutations related to transcription and membrane-associated proteins (Table 3) may be adaptive responses to ethanol stress.

It should be noted that the mutations presented in Table 3 include mutations that occurred before *and* after growth adaptation. Since biological changes start to take place as soon as the *rex* gene is successfully deleted, mutations may already have accumulated by the time *rex* deletion colonies were picked and analyzed, thus the pre-adaptation strains Rex 2, 4, 5, 8 may also contain mutations. However, we were unable to sequence the genome of the pre-adaptation strains (Rex 2, 4, 5, 8) because the adaptation happened so quickly—we saw a significant increase in growth rate after only 2–3 serial transfers. To re-grow strains Rex 2, 4, 5, 8 for genome sequencing, we would essentially be introducing a new round of adaptation for faster growth, where new mutations may occur. This may explain the difference in NADH-linked ADH activity in the pre-adaptation *rex* deletion strains (Table 4, Rex 2, 4, 5, 8), as some *adhE* mutations may have already accumulated in subpopulations of these strains. Another possible reason for the difference in ADH activity in pre-adaptation strains is the mutations in biosynthesis genes discussed above. Since *adhE* is one of the most highly expressed genes in *T*. *saccharolyticum* [8], changes in biosynthesis may largely affect the percentage of AdhE proteins in the cell extract, which will in turn affect NADH-linked ADH activity.

### Complementation of the *rex* deletion

When the *rex* gene was restored in strain RexAdp-4, we observed ethanol yield decrease by half, suggesting that Rex was now inhibiting *adhE* expression, and providing additional evidence that the previous increase in ethanol yield (when *rex* was initially deleted) was due to the specific function of the Rex protein. The ethanol yield of RexCmp-4 was lower than what was observed in the wild type strain, which suggests a role for at least one of the secondary mutations. The most likely candidate is the mutation upstream of *adhE* (Table 3), which could also explain the decreased *adhE* expression in the RexAdp-4 strain (Fig 2).

We did not observe a change in ethanol yield when *rex* was restored in strain RexAdp-5, generating strain RexCmp-5. We believe the explanation for this result is that the RexAdp-5 strain (and subsequently RexCmp-5 strain) contained an AdhE mutation that eliminated its ADH activity (Table 4), and thus masked the result of the *rex* complementation.

### Putative Rex-regulated genes

In a previous study, Ravcheev et al. utilized comparative genetics to analyze 119 genomes from 11 bacterial taxonomic groups (not including *T*. *saccharolyticum*) in search of a common set of genes regulated by Rex [16]. Their results predicted that a small set of “core Rex regulons” existed in different organisms, meaning these genes were predicted to be regulated by Rex in at least two taxonomic groups. “Core Rex regulons” included genes in energy metabolism, central glycolytic pathways, and fermentation [16]. In *T*. *saccharolyticum*, our sequence analysis also predicted a number of genes involved in energy metabolism and electron transfer, carbon metabolism, and fermentation (Table 5). In particular, two putative Rex-binding sites were found upstream of *adhE* and one upstream of *adhA* (Table 5), indicating a high degree of control of Rex on *T*. *saccharolyticum* ethanol fermentation [1].

Additionally, Ravcheev et al. presented three Rex regulons specific to the *thermoanaerobacterales* taxon: *hydG*, *echABCDEF*, and *hypABFCDE* [16]. The *thermoanaerobacterales* taxon in the Ravcheev et al. study included three genomes (*T*. *ethanolicus*, *Thermoanaerobacter tengcongensis*, and *Caldicellulosiruptor saccharolyticus*), and putative Rex-binding sites were predicted with the RegPredict Web server tool (regpredict.lbl.gov) [16]. In this study, we utilized a different site-prediction platform PATLOC (http://www.cmbl.uga.edu/software/patloc.html) to find putative Rex-binding sites because the *T*. *saccharolyticum* genome was not available in the RegPredict database. Interestingly, despite the difference in algorithms, our predictions in *T*. *saccharolyticum* also yielded *hydG*, *echABCDEF*, and *hypABFCDE*. In *T*. *saccharolyticum*, *echABCDEF* and *hypABFCDE* are part of the same operon lead by a common promoter region, which included the putative Rex-binding site. The *echABCDEF* genes encode the energy-conserving Ni-Fe hydrogenase involved in electron transfer and H_2_ production[39], and the *hypABFCDE* genes encode proteins related to the maturation of the energy-conserving Ni-Fe hydrogenase [16]. *hydG* is a Fe-hydrogenase maturation protein necessary for assembling the active sites of Fe hydrogenases [40]. These results suggest that Rex may play an important role in *T*. *saccharolyticum* energy metabolism and electron transfer.

However, Rex regulons are highly variable between different species, which might be explained by Rex regulons changing rapidly and adapting to the organisms’ different lifestyles and ecological niches [16]. Our analysis in *T*. *saccharolyticum* provides new putative Rex regulons involved in biosynthesis, membrane-associated transportation and sporulation.

### Deleting *rex* as an engineering strategy for higher ethanol production

One goal of this study was to investigate deleting *rex* as an engineering strategy for increased ethanol production in *T*. *saccharolyticum*. Currently the best ethanol-producing strain of *T*. *saccharolyticum* is LL1049 (a.k.a. M1442 [3]), a strain generated by deleting several genes including *ldh* (lactate dehydrogenase) and *pta-ack* (phosphotransacetylase and acetate kinase). LL1049 produces ethanol at 75%~90% theoretical yield under different fermentation conditions [1,3].

However, it was surprising to see a significant decrease in *T*. *saccharolyticum* growth rate associated with the *rex* deletion (Table 2). This growth defect contrasted sharply with previous studies in other organisms [10,12,14,15,17,21], where the Rex-inactivated strains presented growth curves similar to the wild type strain (under anaerobic fermentation conditions). The reduction in *T*. *saccharolyticum* growth may be due to changes in transcription of Rex-regulated growth-related genes, or due to secondary mutations that occurred upon *rex* deletion. When the slow-growing Δ*rex* strains (Rex-2, 4, 5, 8) were selected for faster growth via serial transfers, the resulting strains (RexAdp-2, 4, 5, 8) showed a large decrease in ethanol yield (Fig 1). These results suggest that, contrary to our expectations, we were unable to obtain a strain with both high ethanol yield and robust growth after deleting the *rex* gene. Therefore, deletion of the *rex* gene does not seem to be a promising strategy for improving ethanol yield in *T*. *saccharolyticum*.

## Conclusions

In this work we have demonstrated that the Rex protein plays a role in regulating ethanol production in *T*. *saccharolyticum* by gene deletion and complementation experiments. We identified a number of secondary mutations in *rex* deletion strains that occurred after adaptation for faster growth and correlated with a decrease in ethanol yield. Thus, it appears that in the absence of a functional *rex* gene, there is selective pressure for *T*. *saccharoly*ticum to decrease AdhE activity by introducing mutations within or upstream of the gene. This causes the initial high-ethanol phenotype in *rex* deletion strains to be lost after several rounds of serial transfers. Although deletion of *rex* is not a promising technique for engineering improved ethanol yield, this work sheds light on the role of Rex in regulating ethanol production in this organism.

## Supporting information

S1 Fig Putative Rex-binding motif in *T*. *saccharolyticum*. Locations of putative Rex-binding motifs: -35 bp upstream of *adhA*, -151 bp upstream of *adhE* and -32 bp upstream of *adhE*. Consensus sequence: NTTGTTANNNNNNTAACNNN. Nucleotide coloring: A-red, T-green, G-yellow, C-blue.
(PDF)

S2 Fig Sequence conservation of AdhE. Partial sequence alignment of AdhE from *Thermoanaerobacter ethanolicus*, *Thermoanaerobacter mathranii*, *Thermoanaerobacterium saccharolyticum*, *Entamoeba histolytica*, *Escherichia coli*, *Clostridium thermocellum*, *Leuconostoc mesenteroides*, *Lactococcus lactis*, *Oenococcus oeni*, *and Streptococcus equinus*. Residues highlighted in red are the most conserved; residues highlighted in blue are least conserved. Residues enclosed in blue brackets indicate mutation sites shown in Table 3; mutations include T597K, T597I, and T605I.
(PDF)

S1 Table Fermentation end product analysis. (PDF)

S2 Table qPCR primers. (PDF)
