# Supplementary material for: The redox-sensing protein Rex modulates ethanol production in Thermoanaerobacterium saccharolyticum
Source: PLoS One. 2018 Apr 5;13(4):e0195143. doi: 10.1371/journal.pone.0195143 (PMC5886521; doi:10.1371/journal.pone.0195143)
Supplement: S1 Fig — Locations of putative Rex-binding motifs: -35 bp upstream of adhA, -151 bp upstream of adhE and -32 bp upstream of adhE. Consensus sequence: NTTGTTANNNNNNTAACNNN. Nucleotide coloring: A-red, T-green, G-yellow, C-blue. (PDF) [file pone.0195143.s001.pdf]

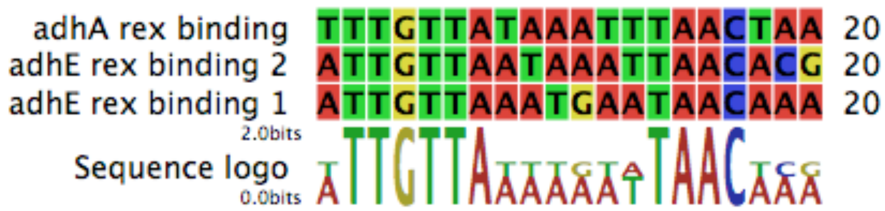

S1 Fig. Putative Rex-binding motif in *T. saccharolyticum*. Locations of putative Rex-binding motifs: -35 bp upstream of *adhA*, -151 bp upstream of *adhE* and -32 bp upstream of *adhE*. Consensus sequence: NTTGTTANNNNNNTAACNNN. Nucleotide coloring: A-red, T-green, G-yellow, C-blue.
