# Supplementary material for: The redox-sensing protein Rex modulates ethanol production in Thermoanaerobacterium saccharolyticum
Source: PLoS One. 2018 Apr 5;13(4):e0195143. doi: 10.1371/journal.pone.0195143 (PMC5886521; doi:10.1371/journal.pone.0195143)
Supplement: S2 Fig — Partial sequence alignment of AdhE from Thermoanaerobacter ethanolicus, Thermoanaerobacter mathranii, Thermoanaerobacterium saccharolyticum, Entamoeba histolytica, Escherichia coli, Clostridium thermocellum, Leuconostoc mesenteroides, Lactococcus lactis, Oenococcus oeni, and Streptococcus equinus. Residues highlighted in red are the most conserved; residues highlighted in blue are least conserved. Residues enclosed in blue brackets indicate mutation sites shown in Table 3; mutations include T597K, T597I, and T605I. (PDF) [file pone.0195143.s002.pdf]

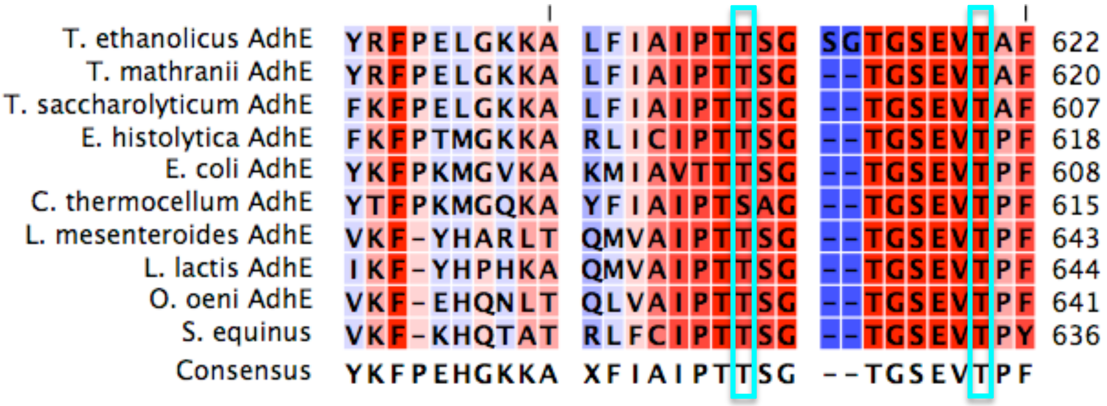

S2 Fig. Sequence conservation of AdhE. Partial sequence alignment of AdhE from *Thermoanaerobacter ethanolicus, Thermoanaerobacter mathranii, Thermoanaerobacterium saccharolyticum, Entamoeba histolytica, Escherichia coli, Clostridium thermocellum, Leuconostoc mesenteroides, Lactococcus lactis, Oenococcus oeni, and Streptococcus equinus*. Residues highlighted in red are the most conserved; residues highlighted in blue are least conserved. Residues enclosed in blue brackets indicate mutation sites shown in Table 3; mutations include T597K, T597I, and T605I.
