# Supplementary material for: The redox-sensing protein Rex modulates ethanol production in Thermoanaerobacterium saccharolyticum
Source: PLoS One. 2018 Apr 5;13(4):e0195143. doi: 10.1371/journal.pone.0195143 (PMC5886521; doi:10.1371/journal.pone.0195143)
Supplement: S1 Table — (PDF) [file pone.0195143.s003.pdf]

S1 Table. Fermentation end product analysis.

Strain[a]	acetate	cellobiose	ethanol	formate	lactate	malate	succinate
	(mM)						
Blank	0.18	14.31	0.00	0.00	0.00	0.00	0.00
Wild type	13.00	0.00	22.66	0.40	12.84	0.04	0.08
Rex2	1.34	0.18	49.50	0.28	1.42	1.88	0.07
Rex4	0.55	0.17	52.90	0.00	0.64	1.48	0.07
Rex5	0.58	0.16	49.03	0.00	2.30	2.10	0.08
Rex8	0.63	0.17	52.13	0.00	0.69	1.46	0.08
RexAdp-2	10.95	0.12	31.51	2.30	0.32	0.62	0.06
RexAdp-4	9.54	0.10	36.55	1.97	2.61	0.53	0.07
RexAdp-5	11.19	0.02	31.79	1.92	0.34	0.23	0.07
RexAdp-8	14.03	0.01	24.98	0.42	6.73	0.26	0.08
RexCmp-4	12.85	0.11	18.94	0.81	19.32	0.21	0.06
RexCmp-5	11.8	0.27	33.73	1.63	1.56	0.7	0.03

[a] The results are the average of biological duplicates
