# Supplementary material for: The redox-sensing protein Rex modulates ethanol production in Thermoanaerobacterium saccharolyticum
Source: PLoS One. 2018 Apr 5;13(4):e0195143. doi: 10.1371/journal.pone.0195143 (PMC5886521; doi:10.1371/journal.pone.0195143)
Supplement: S2 Table — (PDF) [file pone.0195143.s004.pdf]

S2 Table. qPCR primers.

Primer name	Primer sequence	Amplicon sequence
**rex F**	TGGATTTGGACAGCAAGGAT	TGGATTTGGACAGCAAGGATATGGTTACAACGTAGAAGAG<br>CTTTACAATACTTTGACAAAGATTTTAGGTTTAGATAAGA<br>CATACAGCACTATTATCATTGGTGCAGGTAATCTTGGA
**rex R**	TCCAAGATTACCTGCACCAA	
**amp F**	CGCGGTATTATCCCGTATTG	CGCGGTATTATCCCGTATTGACGCCGGGCAAGAGCAACTCG<br>GTCGCCGCATACACTATTCTCAGAATGACTTGGTTGAGTAC<br>TCACCAGTCACAGAAAAGCATCTTACGGATGGCATGACAG
**amp R**	CTGTCATGCCATCCGTAAGA	
**recA F**	GAAGCCTTAGTGCGAAGTGG	GAAGCCTTAGTGCGAAGTGGTGCTGTGGATGTGATCGTTA<br>TTGACTCTGTAGCTGCTCTCGTACCGAAAGCAGAGATAGAT<br>GGTGATATGGGCGATGCACATGTTGGACTTC
**recA R**	GAAGTCCAACATGTGCATCG	
**adhA F**	AGCTCATGGTTTAGGGCTTG	AGCTCATGGTTTAGGGCTTGGTGCAATATTGCCAGCAGTT<br>ATAAAAGCTATTTATCCAGCTACAGCAGAAGTATTGGCTG<br>ATGTATATAGTCCTATAGTTCCTGGTTTAAAAGGACTGCCT<br>GTTGAGGCGGAGTATGT
**adhA R**	ACATACTCCGCCTCAACAGG	
**adhE F**	GCTTCCATCCAAAGGCAATA	GCTTCCATCCAAAGGCAATAAAGTGCAGCATCGCAGCAGCC<br>AAAGTGATGTATGAAGCTGCACTAAAGGCAGGCGCACCTG<br>AAGGATGCATAGGATGGATAGAAACGCCATCAATTGAGG
**adhE R**	CCTCAATTGATGGCGTTTCT	
